# Assessment of Alveolar Bone Status in Middle Aged Chinese (40-59 Years) with Chronic Periodontitis — Using CBCT

**DOI:** 10.1371/journal.pone.0139553

**Published:** 2015-10-02

**Authors:** Haijiao Zhao, Chen Li, Li Lin, Yaping Pan, Hongyan Wang, Jian Zhao, Lisi Tan, Chunling Pan, Jia Song, Dongmei Zhang

**Affiliations:** Department of Periodontics and Oral Biology, School of Stomatology, China Medical University, No.117, Nanjing North Street, Heping District, Shenyang, Liaoning, People’s Republic of China; Second University of Naples, ITALY

## Abstract

**Objective:**

This study used con-beam computed tomography (CBCT) to investigate the prevalence and severity of alveolar bone loss in middle-aged (40–59 years) Chinese with chronic periodontitis.

**Materials and Methods:**

The study group comprised 145 dentate individuals aged 40 to 59 years residing in China who suffered from chronic periodontitis. CBCT and the application of NNT software were used to examine the level and location of alveolar bone loss.

**Results:**

The study revealed that 40–59 year old patients with chronic periodontitis had severe bone loss. At 5,286 sites (34.7%), alveolar bone loss was mild; severe alveolar bone loss was found at 5,978 sites (39.2%). A comparison of bone loss in different jaws revealed that the area with the highest degree of bone loss was on the lingual side of the maxillary molar (56.3 ± 7.2%), and that the area with the lowest degree was primarily on the lingual side of the mandibular canine (27.5 ± 6.3%). There was a lower degree of alveolar bone loss in males than females. Differences were observed when comparing the incidence of bone loss between males and females (*P* < 0.05). Menopause in females and smoking in both genders may affect the level of bone loss. Male smokers experienced a greater degree of bone loss (41.67 ± 5.76%) than male non-smokers (32.95 ± 4.31%). A 42.23 ± 6.34% bone loss was found in menopausal females versus 31.35 ± 3.62% in non-menopausal females.

**Conclusions:**

The study revealed that different sites and teeth exhibited a diverse degree of bone loss. In middle-aged patients with chronic periodontitis, the highest degrees of bone loss in the incisors, premolars, and molars were on the lingual side, mesial side and lingual side, respectively. Menopause in females and smoking may affect the level of bone loss.

## Introduction

In the presence of inflammation, periodontitis develops at a faster rate in the elderly; periodontal lesions also heal with a slower rate in this age group. The prevalence of periodontal disease is particularly high in adults aged ≥ 40 years.[1,2] An epidemiological study[3] conducted with French adults aged 35–64 years reported that clinical attachment level ≥ 5 mm occurred in 46.68%. A study [4] of periodontal tissue in Korean adults revealed that 1.19% of individuals aged 40–59 have > 5 mm clinical attachment loss. Some studies have noted that alveolar bone loss is more prevalent in middle age groups.[5,6] Papapanou et al.[5] found that alveolar bone loss within 3 mm was present in 96% of individuals aged 40–44 years and 95% of individuals aged 50–54 years. Further, periodontal disease is the primary cause of tooth extraction in adults aged ≥ 60 [7, 8]. Therefore, an accurate diagnosis of periodontal disease in the middle-aged population plays an important role in the treatment of the disease and the prevention of future tooth extraction,

The diagnosis and treatment of periodontal disease requires consideration of severity (such as the amount of bone loss) as well as the extent (the percentage or number of affected sites) of the disease.[9] It is difficult to accurately describe the periodontal status of an individual and even more difficult to describe that of a group.

Radiography plays an important role in periodontal diagnosis, primarily because radiographs can reveal the amount and type of damage to alveolar bone which is a major indicator of periodontal disease [10, 11]. Although some studies [12, 13]use periapical and panoramic radiograph imaging procedures to describe the periodontal status of a population, only a few studies have fully assessed buccal and lingual alveolar bone morphology.

In recent years, cone-beam computed tomography (CBCT) has emerged as a feasible tool in dentistry and provides a lower-cost alternative to conventional CT with high-quality images and reduced radiation exposure.[14,15] Furthermore, CBCT has been found to be valuable for the assessment of the degree of bone loss in chronic periodontitis because of its clear and detailed three-dimensional characteristics of alveolar bone morphology, as well as its aid in the diagnosis of the type of bone loss.[16,17] The procedure facilitates the ability of an effective evaluation of periodontal bone injury, especially, crater loss and furcation involvement.

This study used CBCT to evaluate alveolar bone status and the risk factors for this in patients aged 40–59 years with chronic periodontitis. Furthermore, this study provides an objective basis for the formulation of a clinical program.

## Methods

### Study Population

The study was conducted from February 2012 to July 2014 at the Stomatological Hospital of China Medical University. A total of 145 CBCT scans of patients aged 40–59 years were evaluated (females: 69; males: 76). The mean age of the subjects was 46 years; all suffered from chronic periodontitis with periodontal destruction. Periodontal disease status was determined according to clinical and radiographic criteria. All subjects were in good general health and none had received radiation or periodontal treatment during the past six months. None had received antibiotics during the past three months.

No subjects with a history of systemic conditions, such as heart disease, diabetes, or other types of disorders that could influence the course of periodontal disease were enrolled. The enrolled subjects did not use any medications that could affect the manifestations of periodontal disease, such as antibiotics, phenytoin, cyclosporine, anti-inflammatory drugs, or calcium channel blockers.

CBCT images were excluded according to the following criteria: a) unclear visibility of anatomical landmarks, cemento-enamel junction(CEJ), alveolar bone crest(ABC), or tooth apex (AP); or b) visibility of the CEJ was compromised by the presence of restorations, prostheses and other artifacts.

Initially, 4,060 teeth were selected for inclusion in the study. However, some were later excluded from the study. Ultimately, 3,812 teeth (15,248 sides) were included. All recruited patients were apprised of the radiation dose and agreed to imaging studies. Written informed consent was obtained from all of the recruited patients and the study protocol was approved by the local ethics committee of the China Medical University (No-2012-02).

### Clinical Examination

Sites: All subjects underwent pocket depth (PD), clinical attachment level (CAL), bleeding on probing (BOP) and plaque index (PI) measurements. Clinical parameters were measured at four sides per tooth (mesial, distal, buccal, and lingual). The CAL and the probed PD were measured, excluding the third molars. All clinical parameters were measured with a Florida probe (FP32, Version 6; Florida Probe, USA) calibrated in millimeters.

The plaque index (PLI) [18] was scored from 0 to 3, where “0” indicated no plaque. A score of “1” indicated the presence of a plaque film adhering to the free gingival margin and adjacent area of the tooth. The plaque was observed in situ only after using the probe on the tooth surface. A score of “2” represented a moderate accumulation of soft deposits of plaque within the gingival pocket or on the tooth and gingival margin that was visible to the naked eye. A score of “3” represented an abundance of soft matter within the gingival pocket and/or on the tooth and gingival margin.

All clinical data were collected by a single investigator who had been provided with calibration information prior to the beginning of the study. Intra-examiner agreement was evaluated via repeated measurements with a one week interval from the first examination (the intra-examiner kappa value was 0.82).

### Image acquisition

CBCT images of the mandibles were acquired with NEWTOM VG CBCT (QR-NIM s.r.l.; Verona, Italy) using the following acquisition protocol: voxel size 160 μm, acquisition time 26.9 s, tube voltage 110 kV, filament current 5 mA, and field of view 20 ×25 cm.

### Measurement of alveolar bone loss in CBCT images

**Fig 1:**
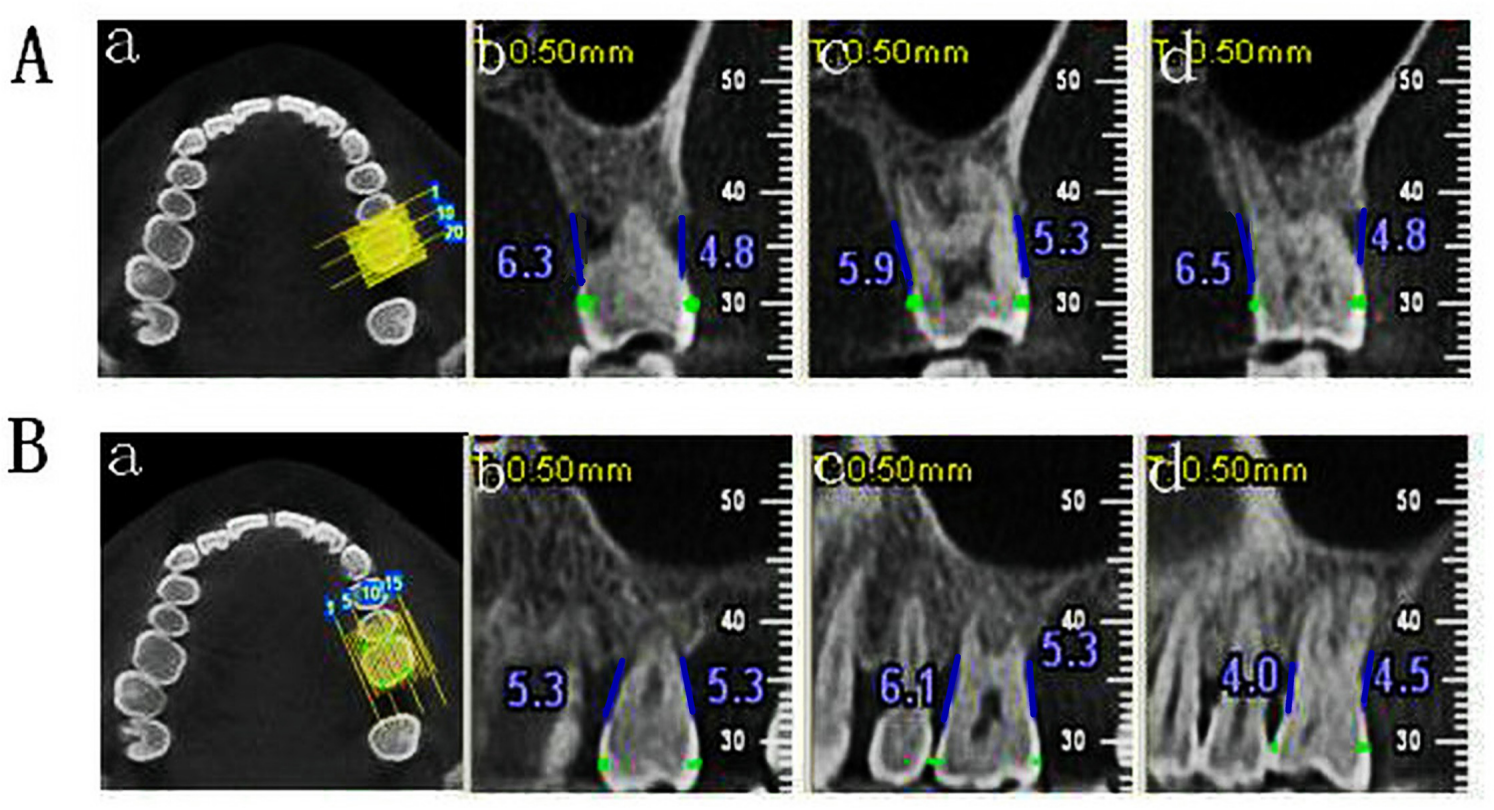
Measurement of alveolar bone loss in CBCT images. A: buccal-lingual cross-section measurement of bone loss of #26; A-a: Mark CEJ of # 26; A-b: BM/LM of #26; A-c: B/M of # 26; A-d: BD/LD; B: Mesio-distal cross-section measurement of bone loss of #26; B-a: Mark CEJ of #26; B-b: ML/DL of #26; B-c: M/D of #26; B-d: MB/DB of #26.

Alveolar bone loss was established when the distance between the CEJ and the ABC was greater than 2 mm. [9] (Fig 1)

**Fig 2:**
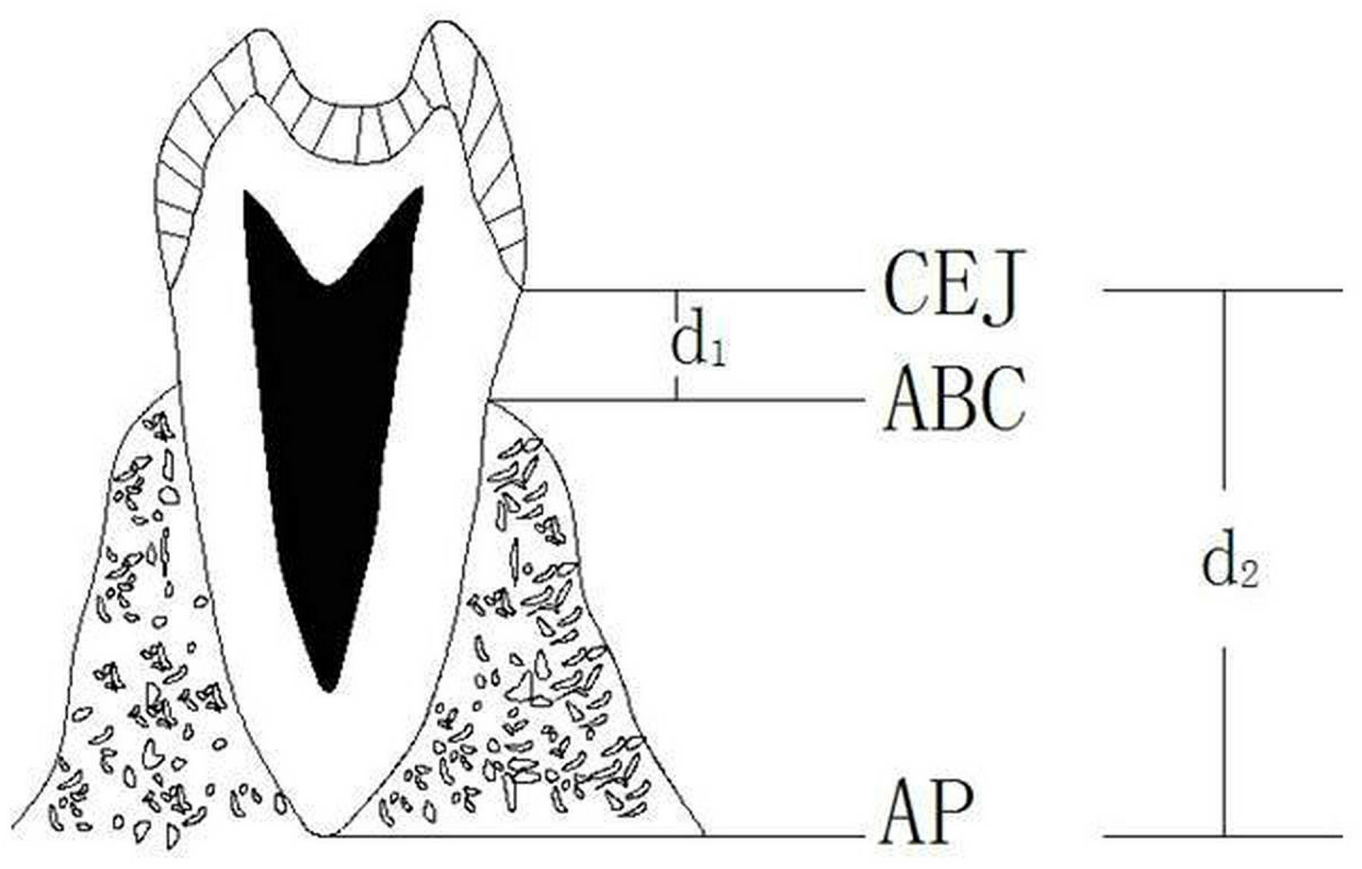
Measurement of alveolar bone loss in patients with periodontitis. d1: Distance from alveolar bone crest to cementoenamel junction; d2: Distance from apical to cementoenamel junction.

The severity of alveolar bone loss was described using the percentage of bone loss. The following linear expressions were then calculated using the distance between the CEJand ABC minus 2 mm/distance between CEJ and AP minus 2 mm:

The percentage of bone loss = (d1–2 mm)/ (d2–2 mm) × 100%.[9] (Fig 2)

**Fig 3:**
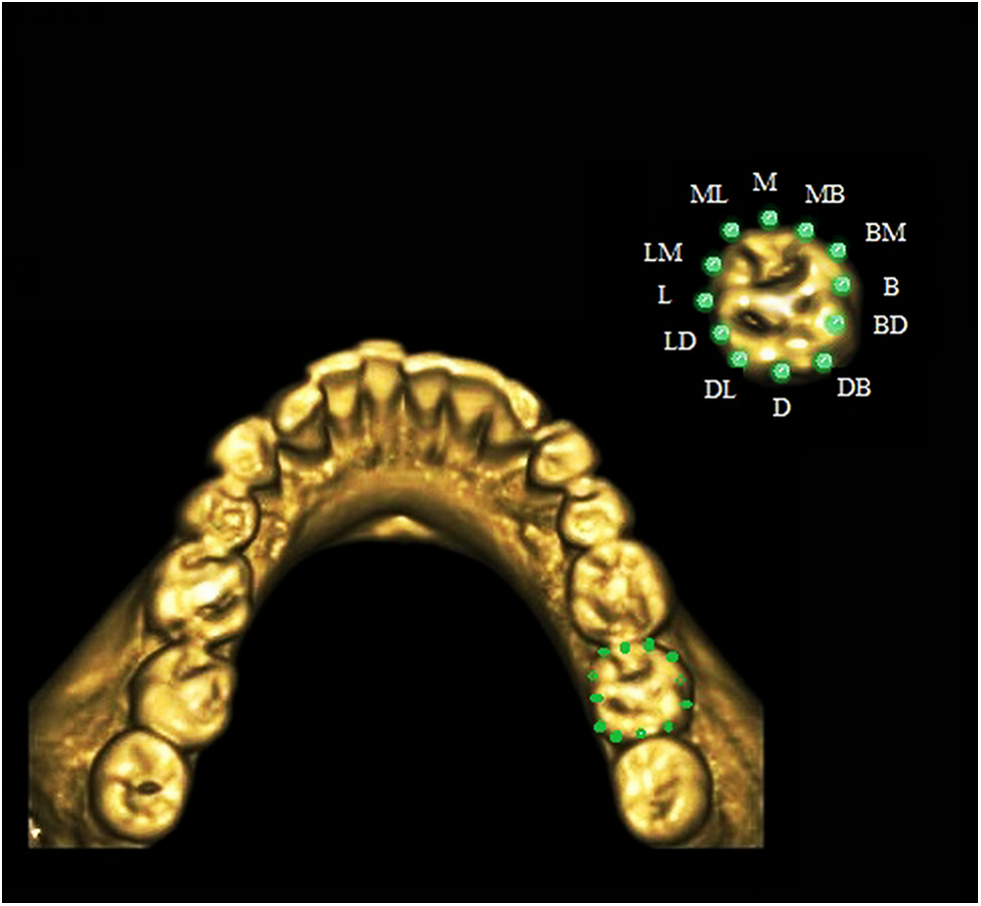
Measurement points. B: buccal; BM: buccal-mesial; BD: buccal-distal; D: distal; DB: distal–buccal; DL: distal- lingual; M: mesial; MB: mesial- buccal; ML: mesial-lingual L: lingual; LM: lingual-mesial; LD: lingual–buccal.

CBCT images were displayed on NNT software (QR-NIM s.r.l.; Verona, Italy). Cross-sectional images with a thickness of 1 mm and an interval of 1 mm perpendicular to the mesiodistal and buccolingual axes of each tooth were prepared. For standardization purposes, there were 12 measurement sites in each tooth: 3 on the buccal aspect, 3 on the palatal aspect, 3 on the mesial aspect, and 3 on the distal aspect. [19] (Fig 3) Calculations of alveolar bone loss were performed on four sides of each tooth (mesial, distal, buccal, and lingual) and make each aspect 3 sites’ mean value as this aspect’s value.

All clinical data were collected by two investigators and inter-examiner agreement was evaluated by means of repeated measurements.

### Classification of alveolar bone loss

For the Asian validation study, the subjects were classified into three categories based on the severity of periodontal disease using the following criteria [20]: (i) mild: pocket depth 3.5–4 mm, clinical attachment loss 1–2 mm, radiographic bone loss ≤ 1/ 3 root length (optional), tooth mobility not detectable; (ii) moderate: 4 mm<pocket depth ≤ 6 mm, clinical attachment loss 3–5 mm, radiographic bone loss between one third and one-half root length (optional), mild mobility; and (iii)severe: pocket depth > 6 mm, clinical attachment loss > 5 mm, radiographic bone loss ≥ 1/2 root length (optional), tooth mobility degree II–III.

### Assessment of the repeatability of inter-and intra-examiners

Prior to the experiment, a statistical analysis conducted by two physicians after consistency test (kappa > 0.85) revealed that the intra-examiner kappa values ranged from 0.81 to 0.83.

## Statistical Analysis

A statistical data analysis was performed with SPSS software (Version 13.0 for Windows; SPSS, Chicago, IL, USA). Inter-observer variation was determined using the paired *t*-test for two-dimensional measurements. Intra-observer measurement variation was determined using paired *t*-tests of the first and second measurements. Categorical variables were used for the descriptive illustration of the percentage of bone loss, categorical variables were used. The Cochran test (Q) was used for comparison between the different teeth sites. Survey multinomial logistic regression models were used to produce weighted population estimates [21]. The dependent variable was the degree of periodontal disease. In the multinomial models, the mild periodontal disease group serves as the reference group for all comparisons. *P*≤ 0.05 was considered to be statistically significant.

## Results

### Distribution of alveolar bone loss

**Fig 4:**
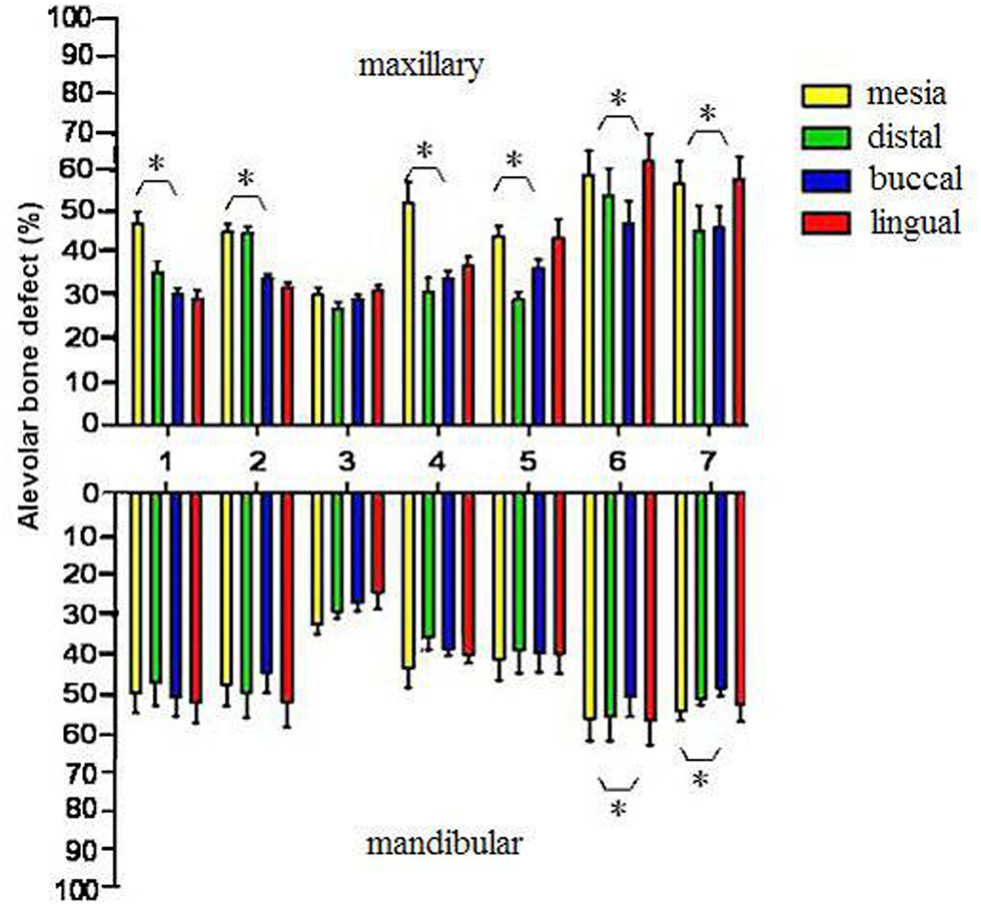
Distribution of alveolar bone loss at different sites. *: Comparison of alveolar bone loss degree on different sides of the same tooth (*P* < 0.05).

A total of 3,812 teeth (15,248 sides) were included in the study. Among these, 3,984 sites (26.1%) had mild alveolar bone loss, 5,286 sites (34.7%) had moderate alveolar bone loss, and 5,978 sites (39.2%) had severe alveolar bone loss (the foregoing sites included incisors and molars with an average degree of alveolar bone loss) (Table 1, Fig 4).

**Table 1:** Comparison of Alveolar Bone Loss in Regard to Different Characteristics (n = number of sites).

	Bone loss (n, %)			
Characteristic	Mild	Moderate	Severe	*P-value*
Gender				0.000
Male	2352 (30.9)	2502 (32.8)	2762 (36.3)	
Female	1632 (21.4)	2784 (36.5)	3216 (42.1)	
Total	3984 (26.1)	5286 (34.7)	5978 (39.2)	

A comparison of bone loss between different jaws revealed that the area with the highest degree of bone loss was the lingual side of maxillary molar (56.3 ± 7.2%) and the lowest site was on the lingual side of the mandibular canine (27.5 ± 6.3%).

There was a statistically significant difference between different sides and teeth types (*P* < 0.001). Fig 4 shows the percentage bone loss at the four aspects (mesial, distal, buccal and lingual) of the same tooth. The study revealed that at the maxillary central/laterals incisors, mesial bone loss was significantly greater than that of the other areas, whereas at the mandibular central/laterals, lingual bone loss was significantly higher than that of the other areas. No significant difference was found between the two aspects of the canine. Alveolar bone loss on the mesial side of first premolar was more severe than that of the other sites. A greater degree of alveolar bone loss was noted on the palatal side of the molar (*P* < 0.05).

**Fig 5:**
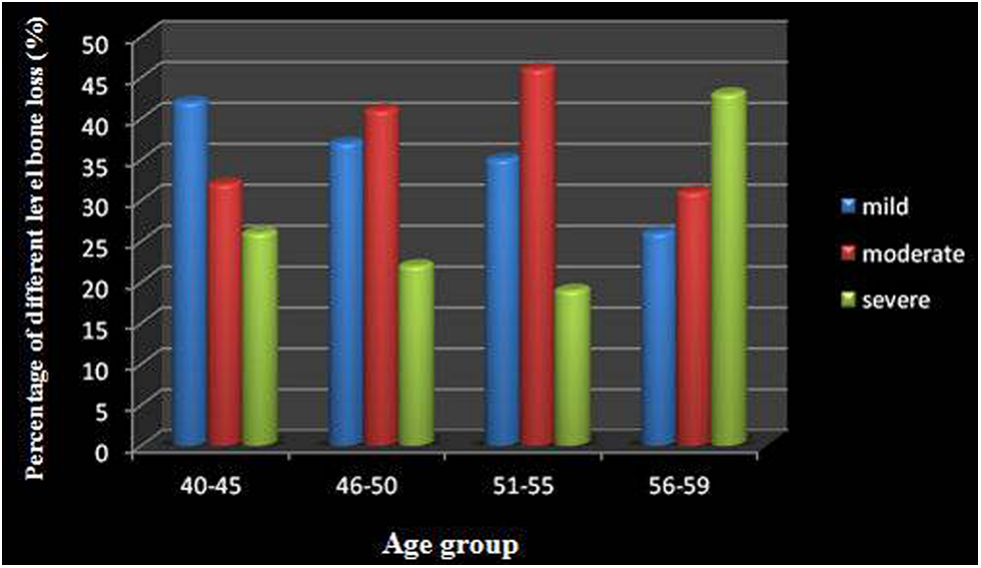
Distribution of bone loss among different age groups.

Fig 5 presents cumulative percentage distributions of age groups according to the degree of bone loss. The younger age groups (age 40–45 years) exhibited more mild bone loss (42.3%), whereas the 46–55 age group exhibited a higher percentage of moderate bone loss (41.5%, 46.1%). The oldest age group (age > 55 years) had the highest percentage of severe bone loss (43.5%).There were significant differences in BOP, PI, PD and CAL between the 40–45, 46–50, 51–55, and 56–59 age groups (*P* < 0.05). (Table 2) The severity of clinical signs increased with age.

**Table 2:** Distribution of BOP, PI, PD, and CAL among individuals in the China population according to gender, age group, smoking and menopause.

		BOP n (%)			PI n (%)					PD ($\bar{\text{x}}$±s) mm		CAL ($\bar{\text{x}}$±s) mm	
		+	_	*P*-value	0	1	2	3	*P*-value		*P*-value		*P*-value
Gender				0.000					0.000		0.021		0.013
	Male	4984 (65.4)	2632 (34.6)		632 (8.3)	1534 (20.1)	1997 (26.2)	3453 (45.3)		3.36±1.16		3.12±1.09	
	Female	5576 (73.1)	2056(26.9)		1175 (15.4)	1962 (25.7)	2013 (26.4)	2482 (32.5)		3.75±1.79		3.89±1.15	
Age group				0.000					0.000		0.000		0.000
	40–45	2148 (53.9)	1836 (46.1)		532 (13.4)	935 (23.5)	1191 (29.9)	1326 (33.3)		2.15±0.67		1.97±0.79	
	46–50	2536 (66.7)	1264 (33.3)		458 (12.7)	923 (25.6)	932 (25.9)	1287 (35.8)		2.98±1.03		2.32±1.25	
	51–55	2792 (75.7)	894 (24.3)		421 (12.1)	873 (25.0)	969 (27.8)	1223 (35.1)		3.56±1.24		3.46±1.07	
	56–59	3084 (81.6)	694 (18.4)		396 (10.5)	765 (20.2)	918 (24.3)	1699 (45.0)		4.95±1.56		4.02±1.53	
Smoking				0.000					0.000		0.000		0.000
	Yes	4221 (89.0)	521 (11.0)		297 (6.3)	809 (17.1)	1226 (25.9)	2410 (50.8)		4.78±1.23		4.25±1.36	
	No	763 (26.5)	2111 (73.5)		335 (11.7)	725 (25.2)	771 (26.8)	1043 (36.3)		2.45±0.96		2.56±1.13	
Menopause				0.000					0.002		0.013		0.008
	Yes	4762 (87.3)	691 (12.7)		834 (15.4)	1375 (25.4)	1392 (25.8)	1802 (33.4)		4.13±1.37		3.57±1.27	
	No	814 (37.4)	1365 (62.6)		341 (15.6)	587 (26.9)	621 (28.5)	630 (28.9)		1.76±1.52		2.05±0.97	

PD: pocket depth, CAL: clinical attachment level, BOP: bleeding on probing, PI: plaque index. (n = number of sites)

### Gender differences

In regard to gender, there was a lower degree of alveolar bone loss in the males (the degrees of bone loss stratified by age group were 30.9%, 32.9%, and 36.3%, respectively). Additionally, there was a higher degree of alveolar bone loss in the females (the degrees of bone loss stratified by age group were 21.4%, 36.5%, and 42.1%, respectively) with statistically significant differences between the groups (*P* < 0.05). (Table 1)

The differences in BOP, PI, PD and CAL were statistically significant between the two sexes (*P* < 0.05). (Table 2)

### Smoking and menopause differences in the degree of bone loss and the clinical parameters of periodontal disease

Table 3 summarizes alveolar bone loss in smokers versus no-smokers. The distribution of alveolar bone loss was 41.67 ± 5.76% in men who smoked and 32.95 ± 4.31% in men who did not (P < 0.05). Statistically significant differences were also found between menopausal women and non-menopausal women (42.23 ± 6.34% bone loss was found in menopausal women versus 31.35 ± 3.62% in non-menopausal women).

**Table 3:** Distribution of Bone Loss in Males by Smoking habit and in Females by Menopausal status ($\bar{\text{x}}$±s).

	Smoking			Menopause		
	Yes	No	*P-value*	Yes	No	*P-value*
Bone loss (%)	41.67±5.76	32.95±4.31	0.000	42.23±6.34	31.35±3.62	0.000

Both the average probe depth and the clinical levels of attachment loss in men who did not smoke were found to be slightly smaller than that of smokers (Table 2). There was a statistically significant difference in the clinical parameters of BOP and PI between smoking and non-smoking individuals. There was also a statistically significant difference in the average probe depth, BOP, PI and the clinical levels of attachment loss between menopausal and non-menopausal women (*P* < 0.001) (Table 2).

### Multinomial regression analysis of the correlations between the degree of periodontal disease and risk factors

In the multinomial regression model (Table 4), gender, age>50 years, smoking and menopause were consistent statistically significant potential risk factors for periodontal disease.

**Table 4:** Multinomial regression analysis of the associations between risk factors and the degree of alveolar bone loss.

	Moderate bone lossOR (95%IC)	Severe bone lossOR (95%IC)
Age		
40–45	1.03 (0.37, 1.52)	1.89 (1.14, 2.35)
46–50	1.58 (0.71, 1.97)	2.12 (1.04, 3.96)*
51–55	1.38 (0.97, 2.93)*	1.96 (0.73, 2.85)*
56–59	2.97 (1.06, 4,31)*	3.86 (2.13, 6.94)*
Gender		
(male = 0,female = 1)	11.18 (0.28, 34.96)*	10.82 (4.78, 24.51)*
Smoking		
(non-smoking = 0,smoking = 1)	1.44 (0.75, 3.69)*	1.95 (1.01, 3.56)*
Menopause		
(non-menopause = 0,menopause = 1)	2.15 (1.07, 9.71)*	4.35 (2.19, 6.51)*

Odds ratios are from a weighted survey multinomial logistic regression.

Participants with mild bone loss are the reference group.

*P≤0.05.

## Discussion

The results of this study suggest that alveolar bone loss is associated with aging. An analysis of 145 patients with chronic periodontitis revealed that 39.2% of 40–59 years old patients had severe alveolar bone loss. Furthermore, the type of tooth, gender, smoking, and menopause appeared to be contributing factors to alveolar bone loss.

In recent years, the degree of alveolar bone loss in middle-aged patients with chronic periodontitis has attracted the attention of researchers. [6, 22, 23] Sarajlić et al [22] found that alveolar bone loss was greater in mandibular anterior teeth of 40–60 years old patients with chronic periodontitis than that of patients aged less than 40 years. This finding was in agreement with our study, which demonstrated that the 40–59 age groups had a greater percentage of severe bone loss (39.2%). Furthermore, we found that the degree of bone defect decreased with age and that more severe bone loss was present in subjects older than 50. This finding is similar to studies conducted in other nations, such as Müller[23], who found that 50% of 50 year old subjects had considerable bone loss (more than 4 mm at 10% or more sites and 6 mm or more at about 5% of sites).

In patients with chronic periodontitis, the degree of alveolar bone loss differed between the types of tooth. Severe alveolar bone loss in patients with chronic periodontitis in this study primarily occurred in the incisors and molars, and less alveolar bone loss occurred in the canines. The findings regarding severe alveolar bone loss were similar to those reported by Sarajlić et al. [22] The reason for this may be due to the earlier eruption of molars and mandibular anterior teeth, teeth position, and molar furcation factors. Our study also found that in the mandibular incisor and molar area (except the palatal side), the extent of alveolar bone loss was higher than that of the maxillary area. Another study found A 6.0 mm mean depth of the angular bony defects and the greatest mean depth in the maxillary anterior area [24]. These differences may be related to race.

A previous study [9] reported that patients who had chronic periodontitis had side-specific bone loss. This finding was similar to ours; patients with chronic periodontitis in different areas of the same tooth had different degrees of alveolar bone loss. The distal side of the incisor had greater alveolar bone loss than the other sides, the mesial side of the premolar had greater alveolar bone loss than the other sides, and the highest degree of alveolar bone loss was on the palatal side of the molar. This may be because the majority of the mesial sides of the premolars have root concavity [25] and because furcation occurs in the molars. Periodontal therapy should be based on issues related to individual teeth and specific side.

In this study, we analyzed associations between the distribution of bone loss and gender, smoking, and menopause in Chinese patients with chronic periodontitis. The data suggested that the type of alveolar bone loss was associated with gender, smoking, and menopausal status. We evaluated 3,812 teeth (15,248 sides); the degree of bone loss was 36.3% in males and 42.1% in females. These findings agree somewhat with data from previous reports. [24,26] Kasaj [26] et al. found that in the 40–60 years age class, 56.1% of alveolar bone defects could be detected in the female subjects versus 59.1% in male subjects. The reason for this may be somewhat related to the fact that younger women are more sensitive to systemic influences and that men are more sensitive to local influences. The findings of the current study support those of previous studies [27]: greater bone loss destruction may occur among menopausal women compared with non-menopausal women. This may be correlated with Chinese women's eating habits such as a small amount of protein intake and rare outdoor activities. Cigarette smoking has long been suspected to be associated with a variety of oral conditions including periodontal diseases. [28, 29] In our study, smokers have a greater extent of bone loss, as well as a higher prevalence of PD and CAL, compared with non-smokers. Similarly, Tomar [30] recently reported that 52.8% of the cigarette smoking population had one or more sites with attachment loss and a pocket depth of 4 mm or more.

Currently, imaging procedures are commonly used for the diagnosis and accurate measurement of alveolar bone loss due to periodontitis. In the two-dimensional images, evaluation of the degree of buccal and palatal alveolar bone loss are compromised due to image overlapping; thus, it is difficult to determine the actual degree of alveolar bone loss.[16] In recent years, due to its low radiation dose and high spatial resolution, three-dimensional image reconstruction CBCT[14,15] appears to be superior to traditional imaging. CBCT also has the lowest error factor (less than 0.25 mm) and can use computer post-processing software in a scanning range reconstruction of any direction. Vandenberghe et al. [31] confirmed the findings of CBCT in the periodontal region areas of furcation with intraoral digital radiographs. In this CBCT study, we clearly imageD the extent of palatal and buccal alveolar bone loss; in patients with chronic periodontitis, alveolar bone loss was the greatest at the palatal side of the maxillary molars and the buccal side of the mandibular molars. Tyndall’s [32] study also showed that the use of CBCT for measuring buccal and lingual alveolar bone conditions can provide accurate, probing-reliable data, and can clearly show the bony plate structure and the destruction of the bony wall. For morphological evaluation of the teeth and bone destruction, CBCT is superior to two-dimensional imaging technology.

It should be noted this study was limited by its sole used of dental CBCT to evaluate bone loss; future studies should explore the ability of CBCT to predict bone density. However, the satisfactory results of the present investigation could serve as guidelines for clinical treatment.

## Conclusions

This in vivo study used CBCT to measure the degree of alveolar bone loss in chronic periodontitis patients. Alveolar bone loss was assessed on different teeth and on different sides of those teeth. CBCT can therefore provide clinicians with a clear understanding of the morphology of alveolar bone loss in chronic periodontitis patients. CBCT can also be used for targeted treatment planning and may be effective for the treatment of chronic periodontitis.

## References

[1] Susin C, Dalla Vecchia CF, Oppermann RV, Haugejorden O, Albandar JM. Periodontal attachment loss in an urban population of Brazilian adults: effect of demographic, behavioral, and environmental risk indicators. J Periodontol. 2004; 75:1033–1041. 15341364 10.1902/jop.2004.75.7.1033

[2] Bouchard P, Boutouyrie P, Mattout C, Bourgeois D. Risk assessment for severe clinical attachment loss in an adult population. J Periodontol. 2006; 77:479–489. 16512763 10.1902/jop.2006.050128

[3] Bourgeois D, Bouchard P, Mattout C. Epidemiology of periodontal status in dentate adults in France, 2002–2003. J Periodontal Res. 2007; 42:219–227. 17451541 10.1111/j.1600-0765.2006.00936.x

[4] Rheu GB, Ji S, Ryu JJ, Lee JB, Shin C, Lee JY, et al Risk assessment for clinical attachment loss of periodontal tissue in Korean adults. J Adv Prosthodont. 2011; 3:25–32. 10.4047/jap.2011.3.1.25 21503190 PMC3076570

[5] Papapanou PN, Lindhe J, Sterrett JD, Eneroth L. Considerations on the contribution of ageing to loss of periodontal tissue support. J Clin Periodontol. 1991; 18:611–615. 1795058 10.1111/j.1600-051x.1991.tb00098.x

[6] Müller HP, Ulbrich M, Heinecke A. Alveolar bone loss in adults as assessed on panoramic radiographs. (II) Multilevel models. Clin Oral Investig. 2005; 9:105–110. 15841404 10.1007/s00784-005-0304-9

[7] Susin C, Oppermann RV, Haugejorden O, Albandar JM. Tooth loss and associated risk indicators in an adult urban population from south Brazil. Acta Odontol Scand. 2005; 63:85–93. 16134547 10.1080/00016350510019694

[8] Anand PS, Kamath KP, Nair B. Trends in extraction of permanent teeth in private dental practices in Keralastate, India. J Contemp Dent Pract. 2010; 11:41–48. 20461323

[9] Fukuda CT, Carneiro SR, Alves VT, Pustiglioni FE, De Micheli G. Radiographic alveolar bone loss in patients undergoing periodontal maintenance. Bull Tokyo Dent Coll. 2008; 49:99–106. 19129684 10.2209/tdcpublication.49.99

[10] Armitage GC. The complete periodontal examination. Periodontology 2000. 2004; 34:22–33. 14717853 10.1046/j.0906-6713.2002.003422.x

[11] Br¨agger U. Radiographic parameters: biological significance and clinical use. Periodontology 2000. 2005; 39:73–90. 16135065 10.1111/j.1600-0757.2005.00128.x

[12] Hou GL, Hung CC, Yang YS, Shieh TY, Tsai CC. Radiographic alveolar bone loss in untreated Taiwan Chinese subjects with adult periodontitis measured by the digital scanning radiographic image analysis method. Dentomaxillofac Radiol. 2003; http://www.ncbi.nlm.nih.gov/pubmed/1277566432:104-108. 10.1259/dmfr/67162332 12775664

[13] Teeuw WJ, Coelho L, Silva A, van der Palen CJ, Lessmann FG, van der Velden U, et al Validation of a dental image analyzer tool to measure alveolar bone loss in periodontitis patients. J Periodontal Res. 2009; 44:94–102. 10.1111/j.1600-0765.2008.01111.x 18973543

[14] Mol A, Balasundaram A. In vitro cone beam computed tomography imaging of periodontal bone. Dentomaxillofac Radiol. 2008; 37:319–324. 10.1259/dmfr/26475758 18757716

[15] Pinsky HM, Dyda S, Pinsky RW, Misch KA, Sarment DP. Accuracy of three dimensional measurements using cone beam CT. Dentomaxillofac Radiol. 2006; 35:410–416. 17082331 10.1259/dmfr/20987648

[16] Jervøe-Storm PM, Hagner M, Neugebauer J, Ritter L, Zöller JE, Jepsen S, et al Comparison of conebeam computerized tomography and intraoral radiographs for determination of the periodontal ligament in a variable phantom. Oral Surg Oral Med Oral Pathol Oral Radiol Endod. 2010; 109: 95–101. 20123402 10.1016/j.tripleo.2009.10.023

[17] Misch KA, Yi ES, Sarment DP. Accuracy of cone beam computed tomography for periodontal defect measurements. J Periodontol. 2006; 77:1261–1266. 16805691 10.1902/jop.2006.050367

[18] Silness J, Loe H. Periodontal disease in pregnancy II. Correlation between oral hygiene and periodontal condition. Acta Odontol Scand. 1964; 22:121–135. 14158464 10.3109/00016356408993968

[19] Feijo CV, Lucena JG, Kurita LM, Pereira SL. Evaluation of cone beam computed tomography in the detection of horizontal periodontal bone defects: an in vivo study. Int J Periodontics Restorative Dent. 2012; 32:162–168. 22754909

[20] Wu X, Offenbacher S, Lόpez NJ, Chen D, Wang HY, Rogus J, et al Association of interleukin-1 gene variations with moderate to severe chronic periodontitis in multiple ethnicities. J Periodontal Res. 2015; 50: 52–61. 10.1111/jre.12181 24690098 PMC4183738

[21] Hyman JJ, Reid BC. Epidemiologic risk factors for periodontal attachment loss among adults in the United States. J Clin Periodontol. 2003; 30: 230–237. 12631181 10.1034/j.1600-051x.2003.00157.x

[22] Sarajlić N, Topić B, Brkić H, Alajbeg IZ. Aging quantification on alveolar bone loss. Coll Antropol. 2009; 33:1165–1170. 20102064

[23] Müller HP, Ulbrich M. Alveolar bone levels in adults as assessed on panoramic radiographs. (I) Prevalence, extent, and severity of even and angular bone loss. Clin Oral Investig. 2005; 9:98–104. 15834742 10.1007/s00784-005-0303-x

[24] Kasaj A, Vasiliu Ch, Willershausen B. Assessment of alveolar bone loss and angular bony defects on panoramic radiographs. Eur J Med Res. 2008; 13:26–30. 18226994

[25] Zhao H, Wang H, Pan C, Jin X. The relationship between root concavities in first premolars and chronic periodontitis. J Periodontal Res. 2014; 49:213–219. 10.1111/jre.12097 23668795

[26] Choël L, Duboeuf F, Bourgeois D, Briguet A, Lissac M. Trabecular alveolar bone in the human mandible: a dual-energy x-ray absorptiometry study. Oral Surg Oral Med Oral Pathol Oral Radiol Endod. 2003; 95:364–370. 12627111 10.1067/moe.2003.119

[27] Sultan N, Rao J. Association between periodontal disease and bone mineral density in postmenopausal women: a cross sectional study. Med Oral Patol Oral Cir Bucal. 2011; 16:440–447. 21196826 10.4317/medoral.16.e440

[28] Paulander J, Wennström JL, Axelsson P, Lindhe J. Some risk factors for periodontal bone loss in 50-year-old individuals. A 10-year cohort study. J Clin Periodontol. 2004; 31:489–496. 15191581 10.1111/j.1600-051X.2004.00514.x

[29] Bahrami G, Wenzel A, Kirkevang LL, Isidor F, Vaeth M. Risk indicators for a reduced marginal bone level in the individual. Oral Health Prev Dent. 2006; 4:215–222. 16961031

[30] Tomar SL, Asma S. Smoking-attributable periodontitis in the United States: findings from NHANES III. National Health and Nutrition Examination Survey. Journal of Periodontology. 2000; 71: 743–751. 10872955 10.1902/jop.2000.71.5.743

[31] Vandenberghe B, Jacobs R, Yang J. Detection of periodontal bone loss using digital intraoral and cone beam computed tomography images: an in vitro assessment of bony and/or infrabony defects. Dentomaxillofacial Radiology. 2008; 37:252–260. 10.1259/dmfr/57711133 18606746

[32] Tyndall DA, Rathore S. Cone-beam CT diagnostic applications: caries, periodontal bone assessment, and endodontic applications. Dent Clin North Am. 2008; 52:825–841. 10.1016/j.cden.2008.05.002 18805231

